# Using proximal remote sensing in non-invasive phenotyping of invertebrates

**DOI:** 10.1371/journal.pone.0176392

**Published:** 2017-05-04

**Authors:** Xiaowei Li, Hongxing Xu, Ling Feng, Xiao Fu, Yalin Zhang, Christian Nansen

**Affiliations:** 1 State Key Laboratory Breeding Base for Zhejiang Sustainable Pest and Disease Control, Zhejiang Academy of Agricultural Sciences, Hangzhou, China; 2 Key Laboratory of Plant Protection Resources and Pest Management, Ministry of Education, Entomological Museum, Northwest A&F University, Yangling, Shaanxi, China; 3 Department of Entomology and Nematology, University of California Davis, Briggs Hall, Davis, California, United States of America; Natural Resources Canada, CANADA

## Abstract

Proximal imaging remote sensing technologies are used to phenotype and to characterize organisms based on specific external body reflectance features. These imaging technologies are gaining interest and becoming more widely used and applied in ecological, systematic, evolutionary, and physiological studies of plants and also of animals. However, important factors may impact the quality and consistency of body reflectance features and therefore the ability to use these technologies as part of non-invasive phenotyping and characterization of organisms. We acquired hyperspectral body reflectance profiles from three insect species, and we examined how preparation procedures and preservation time affected the ability to detect reflectance responses to gender, origin, and age. Different portions of the radiometric spectrum varied markedly in their sensitivity to preparation procedures and preservation time. Based on studies of three insect species, we successfully identified specific radiometric regions, in which phenotypic traits become significantly more pronounced based on either: 1) gentle cleaning of museum specimens with distilled water, or 2) killing and preserving insect specimens in 70% ethanol. Standardization of killing and preservation procedures will greatly increase the ability to use proximal imaging remote sensing technologies as part of phenotyping and also when used in ecological and evolutionary studies of invertebrates.

## Introduction

There is growing understanding of how high-resolution non-invasive acquisition of surface reflectance features from plants and invertebrates can be used to detect and quantify phenotypic responses by individual organisms [1]. That is, the human eye is insufficiently sensitive to detect and quantify subtle phenotypic stress responses to specific factors or treatments, and there is a rapidly growing appreciation for the value and potential of advanced imaging technologies in disciplines, such as, plant and animal phenomics [2–5]. Development of automated and high-throughput systems for plant phenotyping is already under way [6], and we predict that image-based phenotyping of animals will become equally important in the near future as a method to obtain both non-invasive and quantitative data about organismal responses to treatments and environmental factors and genetic make-up. Moreover, we argue that the potential of advanced analyses of surface reflectance features is similar to that of complex studies of genomes and protein profiles as part of addressing fundamental and applied evolutionary and ecological questions.

Proximal remote sensing consists of acquiring and classifying reflectance or transmittance data at one or multiple wavelengths from target objects within a short distance (under 1 m and typically much less) from an imaging sensor [7]. Thus, traditional photography falls under this broad definition provided that the imaging data are analyzed in a quantitative manner as part of detecting or characterizing shapes, features or patterns. Proximal RGB (Red, Green, and Blue spectral bands) remote sensing data have been used to develop 3D models of insects and to conduct automated insect systematics [8]. Proximal RGB remote sensing data from wings and aculeus have also been used to accurately classify closely related species of fruit flies [*Anastrepha fraterculus* (Wied.), *A*. *obliqua* (Macquart), and *A*. *sororcula* Zucchi] [9]. Finally, Proximal RGB remote sensing data have been included into classification studies of bee and lepidopteran species [10–12]. In other applications of proximal remote sensing, data with high spectral resolution (in hundreds of narrow spectral bands) have been used for species identification of: stored grain insects [13–15], two species of fruit flies [16], heliothine moths [17], torticid moth biotypes [18], and cryptic species of ants [19]. In a recent integrative taxonomy study of seven leafhopper species, proximal hyperspectral remote sensing was used successfully to differentiate species within a challenging cryptic species complex [20]. In a study of sound production by mute cicadas, hyperspectral imaging data from the front wing costae were used to describe unique differences in their wing morphology and sound production compared to other cicada species [21].

Proximal hyperspectral remote sensing has also been used in studies of live insects in which surface reflectance data were acquired non-destructively in time series from the body of individual insects. For instance, a recent study describes how the ontogeny of blowfly pupae can be accuratey described on the basis of non-destructive acquisition and analysis of surface reflectance features [22]. Proximal hyperspectral remote sensing has also been used to demonstrate that surface reflectance features acquired from moth eggs parasitized by three different species of a minute egg-parasitoid (*Trichogramma* spp.) can be used accurately to determine which species of *Trichogramma* is developing inside individual moth eggs [23]. Another study has demonstrated that the body reflectance of insects exposed to a killing agent (insecticidal plant extract or entomopathogenic nematodes) changes significantly in certain part of the radiometric spectrum after exposure times, which coincide with the existing knowledge about the time needed for these killing agents to kill their hosts [24]. In other words, the authors argued that surface body reflectance features are closely linked with internal physiological processes and should therfore be of interest to molecular scientists intersted in selecting the optimal time point for when to destructive collect tissue for advanced molecular analyses. Finally, there are examples of proximal remote sensing applications in studies of viability of tree seeds [25, 26] phenotyping of plants [26, 27], and in studies of spiders [28], frogs [29], and fish [30]. Most of the abovementioned examples of proximal remote sensing studies are based on reflectance data acquired within the 400–900 nm wavelength range. However, some studies include reflectance data in the 700–1000 nm wavelength range [31, 32].

Due to the growing interest in use of proximal remote sensing in phenomics, ecological, and evolutionary research of invertebrates, it is important to identify factors affecting the quality of surface reflectance data. In addition, it is important to identify the spectral regions with highest performance in terms of ability to accurately detect phenotypic reflectance responses to gender, age, and origin of conspecific individuals. Even if these variables are the main objective of a given reflectance-based study of organisms, these factors may confound the ability to detect organismal reflectance responses to environmental conditions or treatments. Thus, it is important to obtain further insight into the relative sensitivity of spectral regions to specific factors, such as, gender, origin, age, and time of storage.

In this study, we hypothesized that the ability to detect phenotypic body reflectance features associated with gender, origin, age, and time of storage are influenced by preparation and preservation procedures of insect specimens. To address this hypothesis, we acquired proximal remote sensing data from three insect species: 1) Leafhopper [*Bothrogonia ferruginea* (Fabricius) (Hemiptera: Cicadellidae] specimens from a museum collection, and 2) newly caught western flower thrips [*Frankliniella occidentalis* (Pergande) (Thysanoptera: Thripidae)] and brown planthopper [BPH, *Nilaparvata lugens* (Stal) (Hemiptera: Delphacidae)] specimens. We examined the effect of cleaning stored museum specimens and killing method of fresh insects, and the analysis was based on interpretation of F-values from generalized linear models of variance (glm) of average reflectance data in each of 211 spectral bands from 405–1010 nm. For instance, if average reflectance of males and females in at specific wavelength were non-significantly different when comparing specimens killed and preserved in 90% ethanol but significantly different when specimens were killed and preserved in 70% ethanol, then three conclusions can be drawn: 1) ethanol concentration affects the ability to distinguish between conspecific males and females based on body reflectance in that particular spectral band, 2) 90% ethanol should be used if the goal is to group specimens across gender and use reflectance values in that particular for characterization of organismal response to environmental conditions or a treatment factor, or 3) 70% ethanol should be used if the goal is to determine the gender of specimens and/or include gender as a covariate or factor in characterizations of organismal response to environmental conditions or a treatment factor. In other words, significant F-values within spectral bands were interpreted as indication of high sensitivity to the particular factor, and the goal was to determine spectral sensitivity to gender, age, and origin of insect specimens. We use the results from this analysis to discuss the suitability of spectral ranges for detection of specific phenotypic traits.

## Materials and methods

### Museum specimens of leafhoppers

A total of 83 leafhopper specimens (41 females and 42 males) [*Bothrogonia ferruginea* (Fabricius) (Hemiptera: Cicadellidae] were included, and they originated from the Entomological Museum, Northwest A&F University. We acquired hyperspectral images from all specimens both before and after cleaning. KQ 118 ultrasonic cleaner and distilled water were used to clean specimens, which were transferred individually to Eppendorf tubes (1.5 ml) containing KQ 118 ultrasonic cleaner and rinsed for 20 sec before being transferred to distilled water. This procedure was repeated three times for each specimen. Subsequently, the specimens were dried in a drying oven (DGG-9030A) for 30 min at 35°C. All hyperspectral image data from leafhoppers were acquired from specimens carefully placed in horizontal position and from the dorsal side facing upwards.

### Newly caught specimens of western flower thrips and brown planthoppers

Specimens of western flower thrips [*Frankliniella occidentalis* (Pergande) (Thysenoptera: Thripidae)] specimens were from a lab reared population, which was originally collected from melon (*Cucumis melo* L.) in a greenhouse at the Institute of Vegetables and Flowers, Chinese Academy of Agricultural Sciences, Beijing in 2007. Since establishment, the western flower thrips colony was reared on fresh bean pods in the climate room (27±1°C and 16:8 L:D photoperiod). Brown planthopper [*Nilaparvata lugens* (Stål) (Hemiptera: Delphacidae)] specimens were from lab reared population, which originated from rice fields in Zhejiang Academy of Agricultural Sciences, Zhejiang province, China. For a minimum of 20 generations, this colony has been reared on TN1 rice plants, which is a susceptible variety to brown planthoppers.

### Killing method and preservation time

We acquired hyperspectral images from young (1 to 5 days old) and old (15 to 20 days old) female and male adults of western flower thrips, which had been killed using one of five different methods: 1) CO_2_, 2) freezing at -20°C for 2h, or 3) transferred to 50%, 70% or 90% ethanol for 2h. A total of 176 western flower thrips were imaged with 5–12 replications of all combinations of age, gender, and killing method. In addition, we acquired images from males and females of brown planthoppers, and these were subjected to one of the three killing methods (50%, 70% or 90% ethanol for 2 h). A total of 68 brown planthoppers were imaged with 10–12 replications of all combinations of gender and killing method. To determine the influence of preserve time in 70% ethanol on reflectance data from insect bodies, we acquired hyperspectral images from: 1) female adults of western flower thrips preserved for 2 h, 2 d, 7 d, 14 d, 21 d, and 28 d, and 2) brown planthopper males and females preserved for 2 h, 1 d, 14 d, and 21 d. A total of 140 western flower thrips were imaged with 16–36 replications of all time periods. A total of 88 brown planthoppers were imaged with 10–12 replications of all combinations of gender and time period preserved in 70% ethanol.

### Hyperspectral imaging

All hyperspectral images were acquired from specimens carefully placed in horizontal position and the dorsal side facing upwards. We acquired hyperspectral images under environmental conditions similar to those described in previous studies [22, 24, 33]. We used a push-broom hyperspectral camera (PIKA XC, Resonon Inc., Bozeman, MT, USA) mounted 20 cm above the specimens, and hyperspectral images were acquired with the spatial resolution of about 50 pixels per mm^2^ under artificial lighting (four 15W 12 V light bulbs with two on either side of the lens). The main specifications of the hyperspectral camera were: interface, Firewire (IEEE 1394b), digital output (14 bit), and angular field of view of 7 degrees. The objective lens had a 17 mm focal length (maximum aperture of F1.4), optimized for the near-infrared and visible near-infrared spectra. We acquired reflectance data in 240 spectral bands from 383–1036 nm (spectral resolution = 2.1 nm), but due to concerns about low signal to noise ratio we only included 211 spectral bands from 435–1008 nm. No spectral binning was conducted, so we examined reflectance values in 211 individual spectral bands. During hyperspectral image acquisition, RH was between 30–40% and temperature 19–22°C in the lab. A piece of white Teflon (K-Mac Plastics, MI, USA) was used for white calibration. Reflectance value was referred to proportional reflectance and compared to reflectance obtained from white Teflon.

### Hyperspectral reflectance data analysis

**Fig 1:**
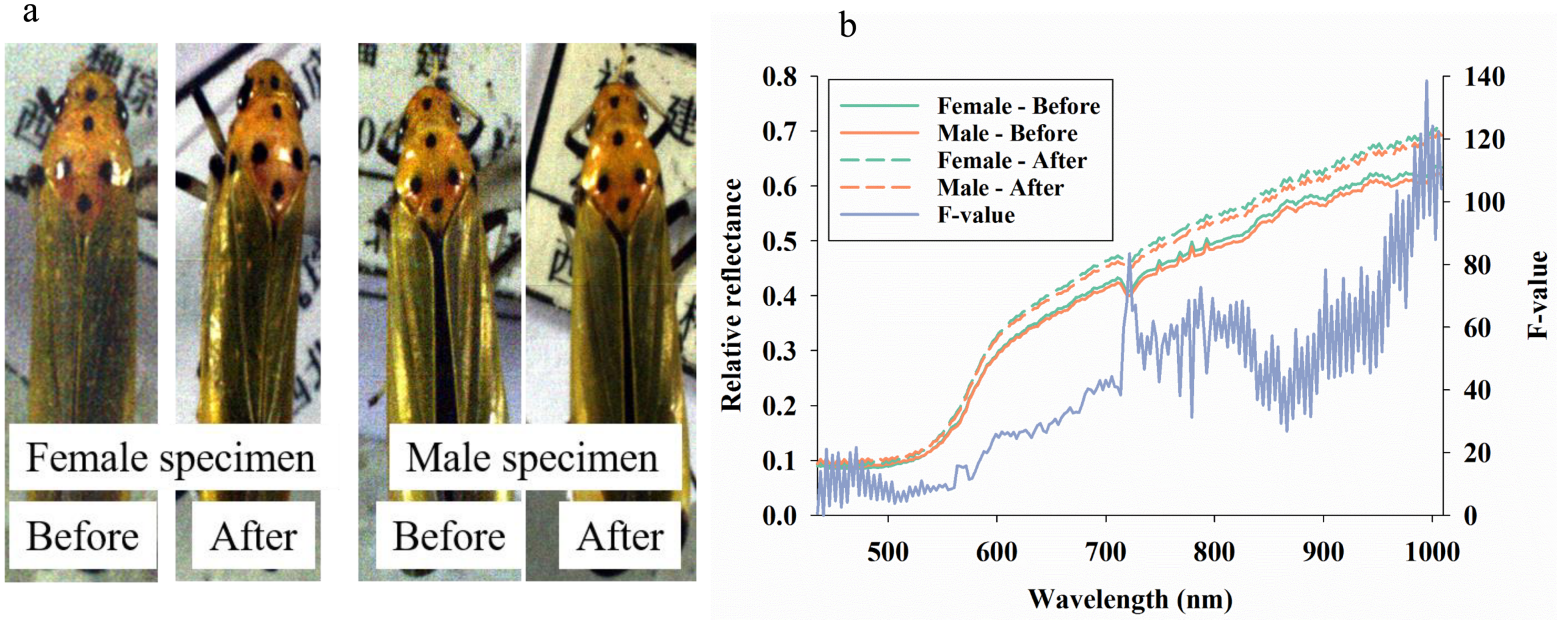
Representative images of leafhopper males and females (*Bothrogonia ferruginea*) before and after cleaning with distilled water (**a**). Average reflectance profiles in 211 spectral bands from 435–1008 nm (**b**). F-values of 211 analyses of variance of the effect of cleaning (across gender) with values exceeding 4.0 being equal to P < 0.05 (dotted black line).

Data processing and analyses were conducted in PC-SAS 9.4 (SAS Institute, NC). For each insect data set, we developed a dichotomous radiometric filter to exclude background (western flower thrips and brown planthopper data sets) and to only include pixels from the orange portion of pronota and head (leafhopper data) (Fig 1). Similarly for western flower thrips and brown planthoppers, white background was excluded through deployment of a dichotomous radiometric filter. Such dichotomous radiometric filters have been used in recently published studies [23, 25, 34, 35]. Based on this data processing step, the average numbers of pixels per specimen were: 1) leafhoppers = 91454 (±517 s.e.), 2) western flower thrips = 40.8 (±1.3 s.e.), and 3) brown planthoppers = 231.4 (±10.6 s.e.).

In the leafhopper data set, we established dummy variables accounting for cleaning (before and after) and gender. We also included a variable accounting for the origin of specimens (province), as the specimens had been collected in five different Chinese provinces. We divided the 83 leafhopper specimens into five year groups, as they had been collected between 1963–2014 [1960 < year < = 1979: year group 1, 1979 < year < = 1989: year group 2, 1989 < year < = 1999: year group 2, 1999 < year < = 2010; year group 4, 2010 < year < = 2015: year group 5]. In the western flower thrips and planthopper data sets, we established dummy variables accounting for gender, and in the western flower thrips data set we also included age [young (1 to 5 days old) and old (15 to 20 days old)] as a treatment factor.

Regarding all three data sets, F-values in 211 generalized linear models of variance (proc glm) of reflectance values in each of 211 spectral bands from 405–1010 nm were used to interpret the relative effect of preparation (cleaning and killing method) on the ability to age, gender, and determine the origin of individual specimens. That is for each of the 211 spectral bands, average relative reflectance values in response to treatments (gender, killing method, and age) were compared. Statistically significant F-values (P < 0.05) indicated a treatment response, so for instance we determined, based on F-values, the difference between males and females both before and after cleaning of museum specimens of leafhoppers. If the F-value of treatment factor “gender” in a spectral was non-significant before cleaning but significant after cleaning, then it could be argued that cleaning increased the ability to separate males and females in that particular spectral band. However, if the F-value of treatment factor “gender” in a spectral was either non-significant or significant both before and after cleaning, then it would indicate low sensitivity to cleaning and therefore high “robustness”. Thus, the significance of F-values can be used to examine the effect of preparation procedures and also to determine which portions of the radiometric spectrum with high levels of robustness to treatment factors. The level of robustness in response to treatment factors is of major importance when developing reflectance-based classification algorithms to study phenotypic responses.

## Results

### Effect of cleaning of museum specimens

Visualization of leafhopper specimens clearly demonstrated the challenge of separating males and females (Fig 1a). In addition, the images of specimens reveal that it is virtually impossible to detect the effect of gentle cleaning with distilled water. In each of 46 spectral bands from 435–558 nm, there was significant effect of cleaning, but the relative effect of cleaning (as expressed by F-values) was markedly higher in spectral bands beyond 700 nm (Fig 1b). The strongest effect on average reflectance (as indicated by F-values) of cleaning was observed at 721 nm and in spectral bands between 950–1010 nm. We found that average reflectance profiles from male leafhoppers were consistently darker than those from conspecific females, both before and after cleaning.

**Fig 2:**
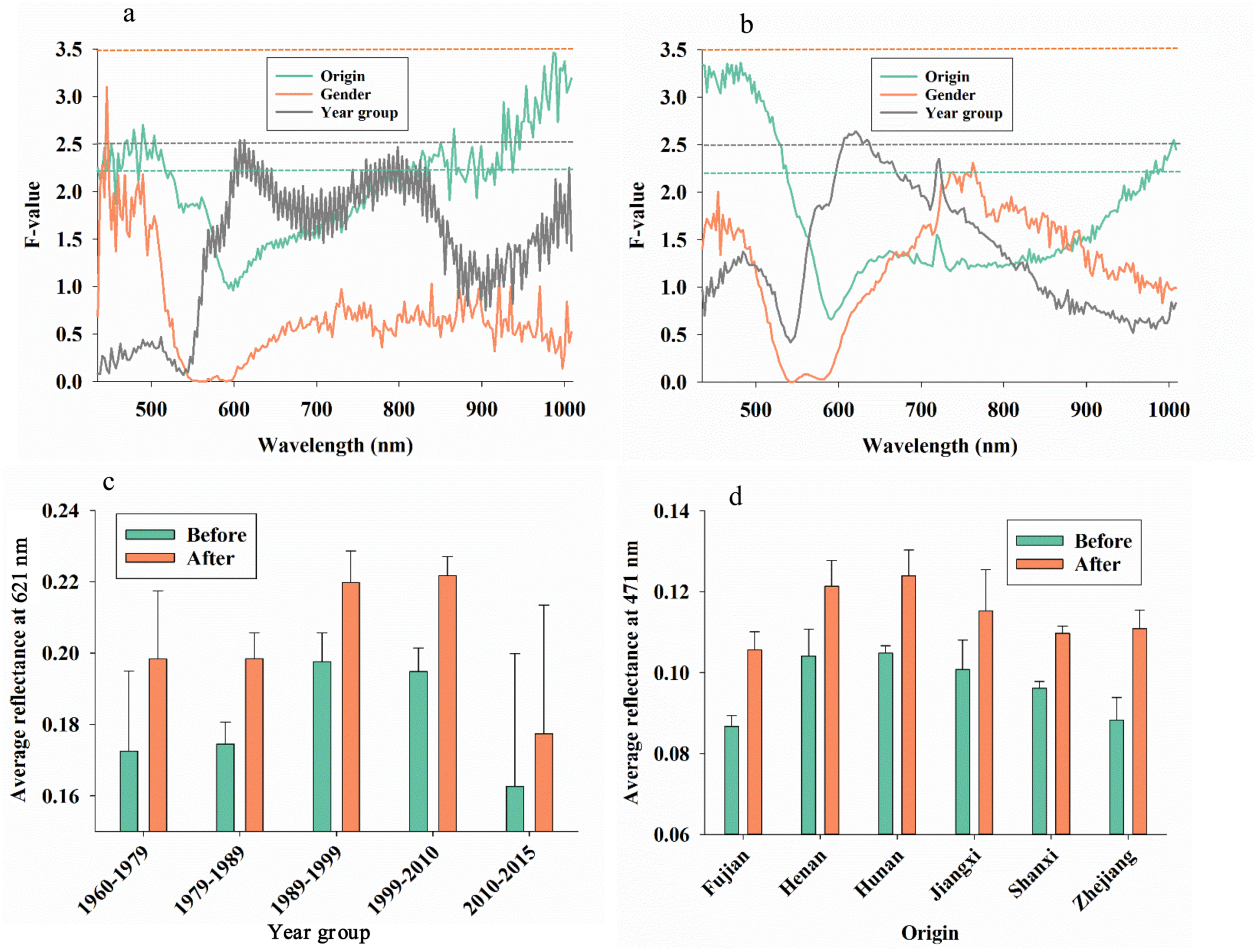
F-values in 211 analyses of variance of average reflectance (211 spectral bands from 435–1008 nm) of leafhoppers (*Bothrogonia ferruginea*) with the following treatment effects before (**a**) or after (**b**) cleaning: origin (province), gender, and year group (years of storage). Due to different degrees of freedom, significance of F-values at the 0.05-level varied for each treatment factor: origin = 2.40, gender = 3.5, and year group = 2.50 (dotted lines with the same colors as treatment factors). Average reflectance at 621 nm (**c**) and 471 (**d**) before and after cleaning in response to year group and origin, respectively.

Before cleaning, we examined the average reflectance response to origin (province), year group, and gender, and we found that (Fig 2a): 1) none of the spectral bands showed significant difference between males and females (in all 211 spectral bands, F-values < 3.5), 2) a few spectral bands around 600 nm showed significant response to year group of specimens (F-values > 2.5), and 3) spectral bands from 408–515 nm and from 830–1008 nm showed significant response to origin (F-values > 2.26). After cleaning, we examined effects of the same factors and found that (Fig 2b): 1) none of the spectral bands showed significant difference between males and females, 2) spectral bands from 605–635 nm showed significant difference among year groups, and 3) spectral bands from 408–540 nm and from 980–1010 nm showed significant response to origin (F-values > 2.26).

Comparing results from before (Fig 2a) and after (Fig 2b) cleaning, it is seen that especially the response to origin of specimens was more pronounced after cleaning, while detection of gender and year groups were less affected by cleaning. To illustrate the average reflectance responses to year group (reflectance at 621 nm), it is seen that cleaning resulted in 10–15% increase in reflectance, irrespectively of year group, and that the most recent specimens had slightly lower reflectance at 621 nm than older specimens (Fig 2c). To illustrate the average reflectance responses to origin (reflectance at 471 nm), it is seen that cleaning resulted in 6–15% increase in reflectance, irrespectively of origin, and that specimens from Fujian and Zhejiang had lower reflectance at 471 nm than conspecific specimens from Hunan and Henan (Fig 2d).

### Effect of killing method of western flower thrips

**Fig 3:**
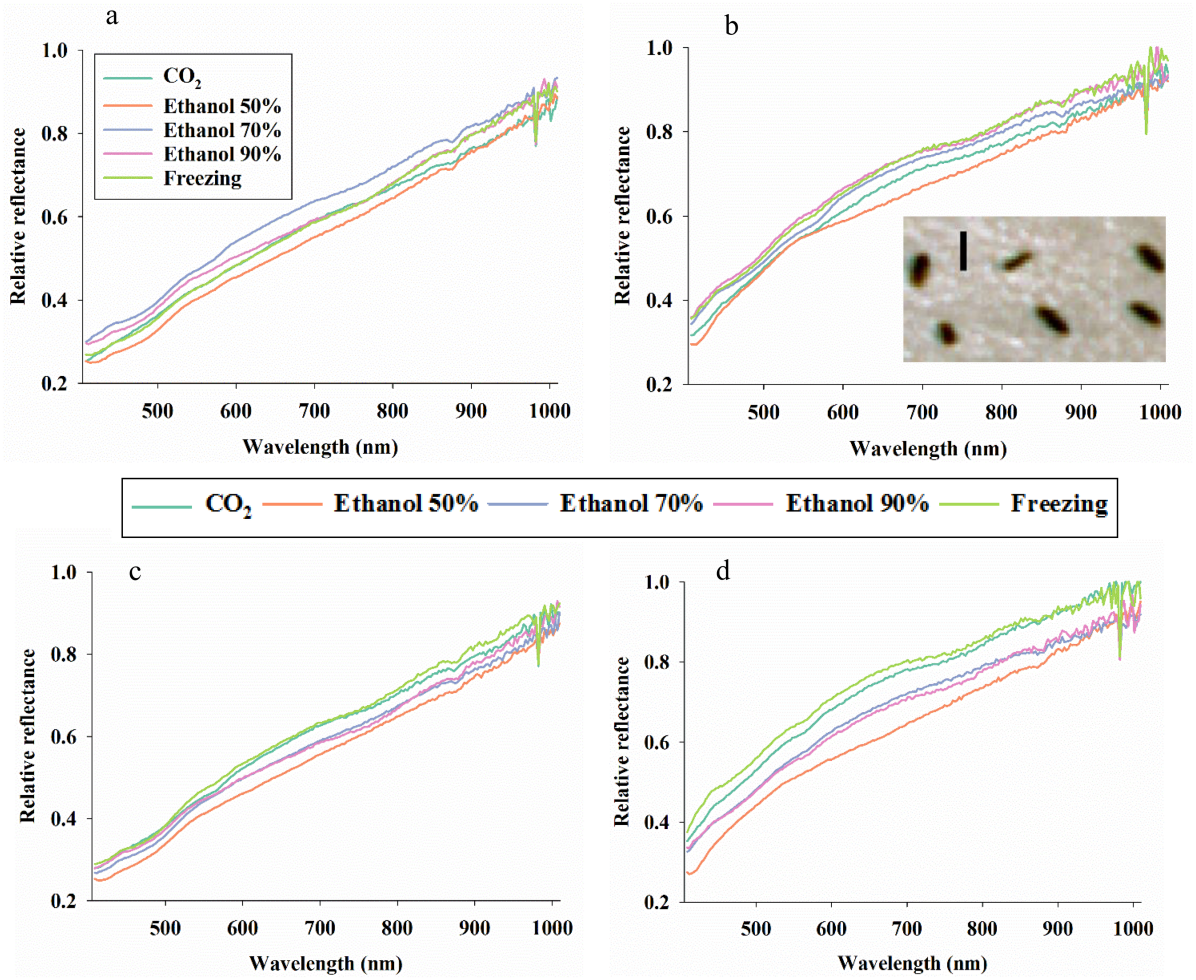
Average reflectance profiles of young females (**a**), young males (**b**), old females (**c**), and old males (**d**) of western flower thrips (*Frankliniella occidentalis*) in 211 spectral bands from 435–1008 nm after killing by five different methods. A representative image of the western flower thrips is included (**b**) with the vertical bar = 1 mm.

As general trends in average reflectance profiles from the four combinations of age and gender of western flower thrips, it is seen that (Fig 3): 1) freezing was associated with low reflectance values (darkest insect body reflectance), 2) CO_2_ was associated with high reflectance values across the examined spectrum, and 3) average reflectance profiles acquired from males were slightly curved with an inflection point around 700 nm, while average reflectance profiles from conspecific females were more straight. The difference in shape of average reflectance profiles from males and females indicate a strong possibility of accurate reflectance-based differentiation of males and females.

**Fig 4:**
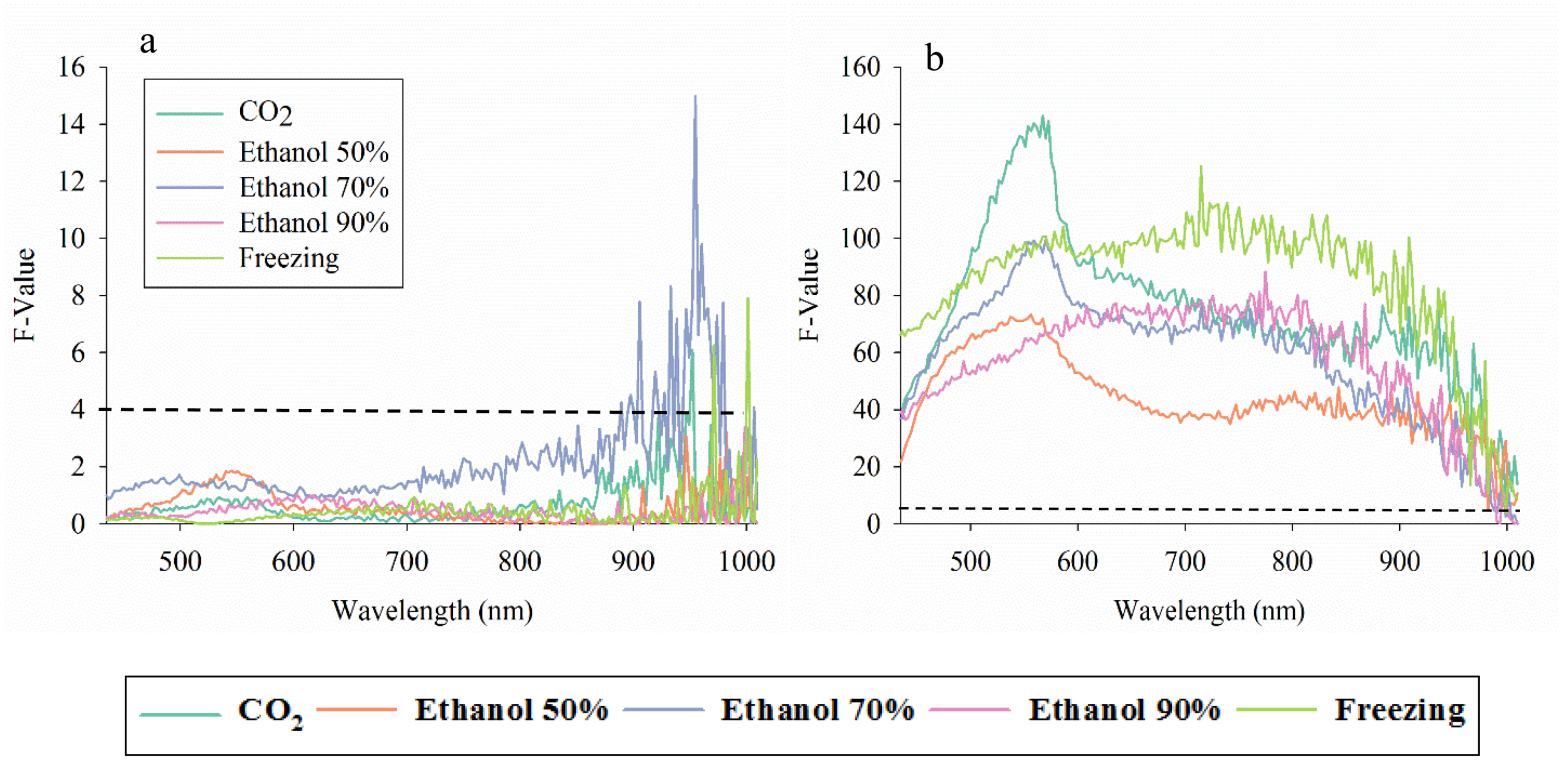
F-values in 211 analyses of variance of average reflectance (211 spectral bands from 435–1008 nm) of western flower thrips (*Frankliniella occidentalis*). We examined effects of five killing methods on the ability to detect reflectance response to age (young versus old) (**a**) and gender (**b**). F-values exceeding 4.0 were significant at the 0.05 level (black dotted line).

For each of the five killing methods applied to western flower thrips, we examined the ability to differentiate between young and old specimens, and none of the spectral bands from 405–900 nm showed a significant response to age, but several spectral bands between 900–1010 nm (i.e. a peak at 954 nm) responded strongly to age, especially for western flower thrips in ethanol 70% (Fig 4a). In the analyses of effect of killing method on the ability to differentiate males and females, we obtained highly significant F-values in all combinations of killing methods and spectral bands from 408–900 nm (Fig 4b). Most significant reflectance response was observed with CO_2_ or freezing and especially in spectral bands near 550 nm. However, there was also a very strong and consistent reflectance response to gender, when 70% ethanol was used as killing method and when examining spectral bands from 600–850 nm.

We examined the effect of preserving western flower thrips females in 70% ethanol for 2 h-28 d, and none of the spectral showed a significant effect (P > 0.05). Thus, it was concluded that all spectral bands were unaffected by storage up to 4 weeks in 70% ethanol.

### Effect of killing method of brown planthoppers

**Fig 5:**
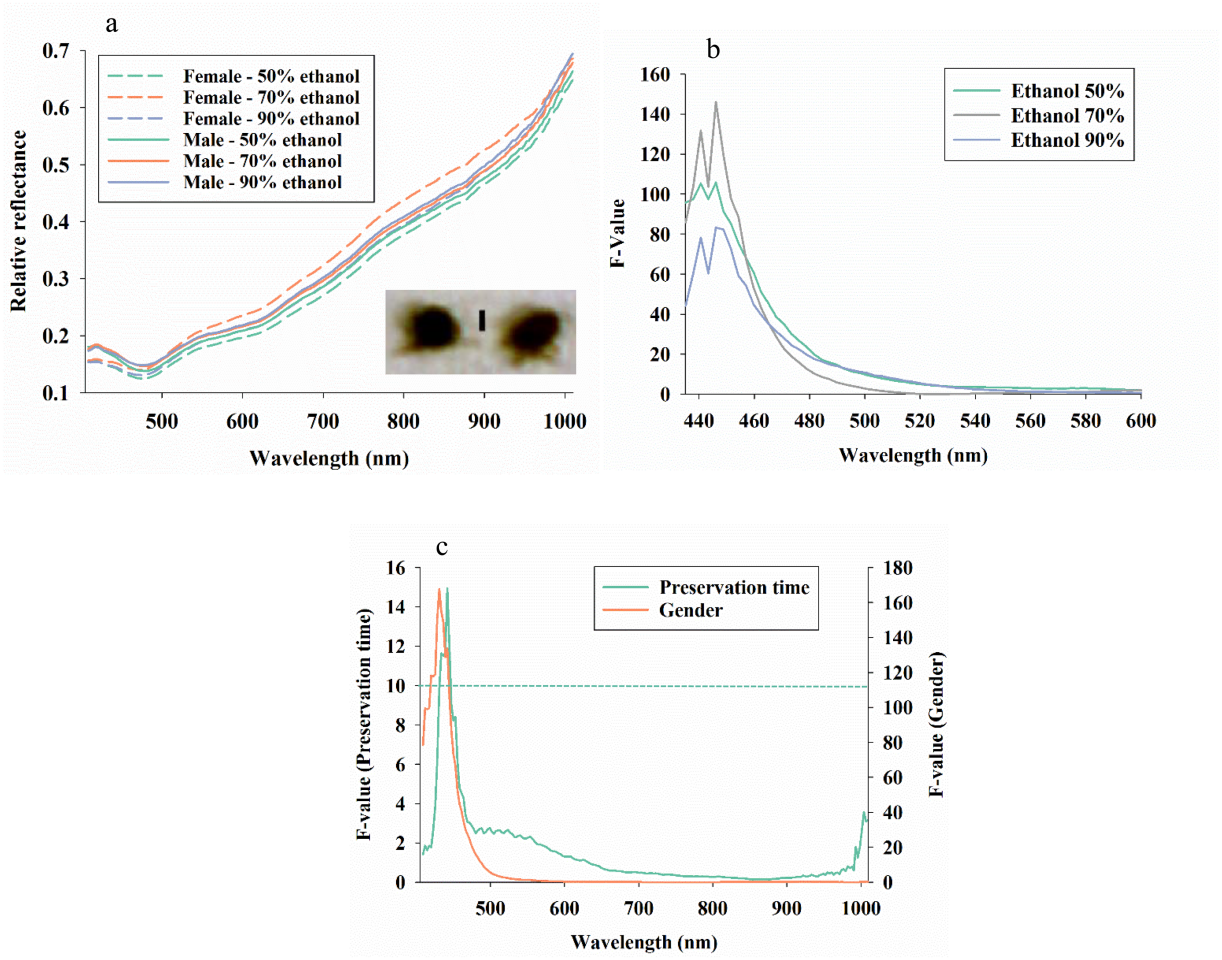
Average reflectance profiles of females and males of brown planthoppers (*Nilaparvata lugens* when killed based on three methods (**a**). A representative image of the brown planthoppers is included (**b**) with the vertical bar = 2 mm. F-values in 211 analyses of variance of average reflectance (211 spectral bands from 435–1008 nm) for each of the three killing methods and with gender as the treatment factor (**b**). F-values exceeding 4.0 were significant at the 0.05 level. F-values in 211 analyses of variance of average reflectance (211 spectral bands from 435–1008 nm) of brown planthoppers killed with 70% ethanol and with gender and preserve time as the treatment factors (**c**). Due to different degrees of freedom, significance of F-values at the 0.05-level varied for each treatment factor: gender = 4.0, preserve time = 10.0 (dotted lines with the same colors as treatment factors).

In the comparison of average reflectance profiles in response to ethanol concentration and gender of brown planthoppers, it is seen that (Fig 5a): 1) males and females had very similar reflectance profiles and 2) ethanol concentration only caused subtle changes to average reflectance values in all spectral bands. We examined the relative effect of ethanol concentration on the ability to differentiate male and female brown planthoppers, and the strongest response was observed in spectral bands from 405–480 nm, especially from insects preserved in 70% ethanol (Fig 5b). F-values in spectral bands beyond 600 nm are not shown in Fig 5b, but they were all very close to zero and therefore suggesting that none of those spectral bands could be used to detect gender, irrespectively of ethanol concentration. Finally, we examined the relative effect of time kept in 70% ethanol (Fig 5c), and only spectral bands from 428–455 nm showed a significant response. Thus, all spectral bands outside the 428–455 nm range would be suitable for classification of gender and other treatments factors irrespectively of preserve time up to 3 weeks in 70% ethanol.

## Discussion

The main goals with this study were to demonstrate the potential power of organismal description (such as gender, age and origin) based on proximal remote sensing. In this regard, the current study is similar to studies of reflectance-based studies of seed viability [25, 26], ontogeny of blowfly pupae [22], and terminal stress response in insects exposed to killing agents [24]. In addition, we showed how important factors may influence the quality of proximal remote sensing data and that 70% ethanol appears to be the most suitable killing method and solvent for preservation over time of killed specimens. We demonstrated that subtle cleaning of museum specimens enabled identification of origin-related reflectance features, and these features were not detectable prior to cleaning. Thus, our analysis emphasizes the importance of subtle cleaning with distilled water, if proximal remote sensing is used in studies of specimens, which have been stored for long periods of time and/or under conditions where dust may deposit on the examined specimens. In the comparison of killing methods, we found that 70% ethanol was generally the method enabling the strongest response to gender of insects, so we recommend this killing method when live organisms are collected from either field or laboratory studies. Finally, we demonstrated that storage of insect specimens in 70% ethanol for several weeks had negligible effect on reflectance data acquired from western flower thrips and only affected reflectance data in the UV-blue light spectrum from brown planthoppers. This result is very important, as it suggests that immediate imaging of specimens is not necessary. Consequently, our results suggest that specimens sampled under field conditions can be safely preserved in 70% ethanol for several weeks before being subjected to proximal remote sensing.

Regarding the importance of spectral ranges for the detection of treatment effects, we found that distinct differences both among factors and among the insect species. Origin of leafhopper specimens was most pronounced in spectral bands from 408–540 nm and 980–1010 nm. In a study of seven cryptic leafhopper species, Wang, Nansen (20) used a combination of 37 spectral bands from 411–870 nm to develop a reflectance based classification algorithm, which was associated with 91.3% accuracy across the seven species. In a study of two closely related stored grain species (*Sitophilus spp*.) and with specimens collected from different regions in China, Cao, Zhang [36] examined hyperspectral reflectance features and identified spectral bands at 505, 659 and 955 nm as contributing the most to their classification algorithms. From this brief review, it appears that detection of spectral ranges associated with strong responses to origin of specimens, biotypes, and species vary considerably among clades of organisms. In addition, we suspect that detection of such spectral ranges with strong response to origin or biotypes is highly influenced by the data classification method, by data filtering steps, and by data processing procedures prior to the development of classification algorithms. Another important factor is the wavelength range being examined, and Mielewczik, Liebisch (32) provided an overview of 180 insect species’ reflectance profiles in the NIR (700–1000 nm) wavelength range. In addition, the study by Mielewczik, Liebisch (32) demonstrated that age and gender of live specimens of locusts could be determined based on reflectance data.

With respect to gender classification, we showed that leafhopper specimens could not be accurately classified, there was a strong gender response of western flower thrips in spectral bands from 600–850 nm, and the gender response of brown planthoppers was strongest in spectral bands from 405–480 nm. Thus, we obtained three very different results with the three species, and we suspect that successful detection of spectral ranges with a strong gender response will likely vary considerably among species.

As an ecological and evolutionary research tool, proximal remote sensing has a number of advantages: 1) Efficient—reflectance or transmittance data can be acquired within a few seconds and no or limited additional preparation (i.e. placing target objects in a specific position) of the target objects is required. 2) Non-destructive—proximal remote sensing data can be acquired from both dead and live plants and animals without any damage to tissues. 3) Quantitative phenotyping—hyperspectral reflectance features are digital data, which are easy to either store or import into statistical software packages. Often, phenotyping is based on qualitative grading/scoring of responses, and that may pose challenges related to training of personnel performing the evaluations and about repeatability. Data handling—although hyperspectral imaging data sets are often large, they are easily sent via the internet to large computers and/or to facilities with advanced data processing and classification capabilities. Robustness and sensitivity—it is equally important that data classification methods are accurate when applied to different data sets (high level of robustness) and at the same time show high level of sensitivity by being able to accurately detect very subtle phenotypic responses. Many applications of proximal remote sensing confirm the high levels of robustness [37] and sensitivity [24, 25] of classifications based on analyses of hyperspectral reflectance features. Cost-effective—after the initial purchase of equipment, proximal remote sensing systems only requires minor maintenance. These advantages underscore the potential of proximal remote sensing technologies as part of ecological and evolutionary research.

Although this study only involved insect species, the results have broader applications, as proximal remote sensing technologies are also applied to studies of spiders [28], frogs [29] and fish [30]. Standardized killing and preservation of specimens in 70% ethanol was identified as the “best practice” regarding killing and preservation of fresh specimens, as we obtained the strongest response (highest F-values) to gender, age, and origin with this killing method. As the understanding of the associations between reflectance features and physiological and genetic traits increases, and proximal remote sensing technologies therefore become more widely used in ecological and evolutionary studies, it becomes progressively more important to standardize both reflectance data acquisitions and the preparation procedures of organisms being imaged.

## Supporting information

S1 Table Raw data of relative reflectance of leafhopper females and males (*Bothrogonia ferruginea*) collected from different provinces in different years in 211 spectral bands from 435–1008 nm. Fem: female; Mal: male; 0: before cleaning; 1: after cleaning; B10-230: spectral band 10 to band 230.
(XLS)

S2 Table Raw data of relative reflectance of western flower thrips (*Frankliniella occidentalis*) in 211 spectral bands from 435–1008 nm after killing by five different methods. Fem: female; Mal: male; You: young thrips (1 to 5 days old); Old: old thrips (15 to 20 days old); CAR: killed by CO_2_; E50: killed in 50% ethanol; E70: killed in 70% ethanol; E90: killed in 90% ethanol; FRE: killed by freezing at -20°C for 2h; B10-230: spectral band 10 to band 230.
(XLS)

S3 Table Raw data of relative reflectance of western flower thrips females (*Frankliniella occidentalis*) in 211 spectral bands from 435–1008 nm preserved in 70% ethanol for different hours. Fem: female; E70: preserved in 70% ethanol; B10-230: spectral band 10 to band 230.
(XLS)

S4 Table Raw data of relative reflectance of brown planthoppers females and males (*Nilaparvata lugens*) in 211 spectral bands from 435–1008 nm when killed based on three methods. Fem: female; Mal: male; E50: killed in 50% ethanol; E70: killed in 70% ethanol; E90: killed in 90% ethanol; B10-230: spectral band 10 to band 230.
(XLS)

S5 Table Raw data of relative reflectance of brown planthoppers females (*Nilaparvata lugens*) in 211 spectral bands from 435–1008 nm preserved in 70% ethanol for different hours. Fem: female; E70: preserved in 70% ethanol; B10-230: spectral band 10 to band 230.
(XLS)
